# Improvement of the Thermoelectric Power Factor of ZnO Using Ionic Liquids

**DOI:** 10.1021/acsaelm.5c01462

**Published:** 2026-01-29

**Authors:** Md Mahmudur Rahman, Lourdes Márquez-García, Guillem Montaña-Mora, Ke Xiao, Mauricio Solis-de la Fuente, Sergio Castro-Ruiz, Sébastien Fantini, Andreu Cabot, Jorge García-Cañadas

**Affiliations:** † 16748 Universitat Jaume I, Av. Vicent Sos Baynat s/n, Castelló de la Plana 12003, Spain; ‡ Institut de Recerca en Energia de Catalunya (IREC), Jardins de les Dones de Negre 1, Sant Adrià de Besòs, Catalonia 08930, Spain; § 374981 Solvionic, 11 Chem. des Silos, Toulouse 31100, France; ∥ ICREA, Pg. Lluis Companys, Barcelona, Catalonia 08010, Spain

**Keywords:** hybrid solid–liquid thermoelectric, ZnO, ionic liquid, iodide, impedance spectroscopy

## Abstract

Thermoelectric (TE) materials are able to convert heat into electricity. Since many heat sources are available in our sourroundings (e.g., body heat, sun, domestic heat), thermoelectricity have drawn significant interest for energy harvesting. The power factor (PF = *S* ^2^σ, being *S* and σ the Seebeck coefficient and the electrical conductivity, respectively) and the thermal conductivity are key parameters to assess materials’ performance. Our group reported a few years ago a concept to significantly improve the PF. It was based on the combination of a porous TE solid with a liquid electrolyte. PF improvements above 3 times were achieved when Sb-doped SnO_2_ was used as the solid. However, despite this substantially high PF enchancement, not very high PFs were reached due to the modest TE properties of Sb/SnO_2_. Here, we aim at introducing similar PF improvements in a high-performance porous TE oxide, a Ag–ZnO composite, by means of its combination with different ionic liquids acting as electrolytes: 1-butyl-3-methylimidazolium iodide (BMII) and 1-butyl-3-methylimidazolium bis­(trifluoromethylsulfonyl)­imide (BMITFSI). For the BMII ionic liquid, it was found a significant increase (70.2%) in σ with only a small variation in *S*. This uncommon and highly befenefitial disconnection of *S* and σ, which typically are adversely related, led to a PF improvement of 1.46 times. In contrast, samples contacted with BMITFSI showed almost no PF variation. Scanning electron microscopy, X-ray diffraction, and impedance spectroscopy experiments showed that the electrical conductivity increase is due to a rise in the carrier concentration in the oxide, produced by the injection of electrons from the iodide ions. Hence, our work demonstrates that the strategy of enhancing the PF using electrolytes can also be extended to ZnO, one of the best-performing TE oxides.

## Graphical abstract

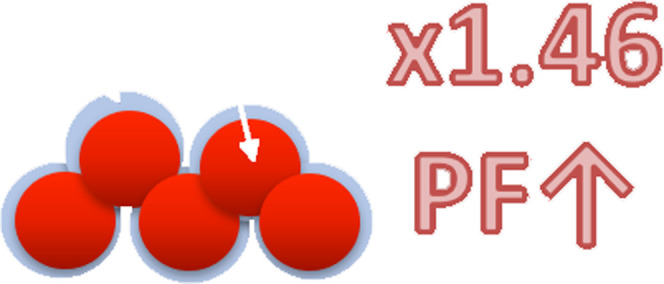

## Introduction

Currently, around 72% of the total global energy consumption is lost as waste heat.^1^ Only a 10% recovery of this heat would surpass the summation of the energy generated by the current sustainable energy sources (solar, wind, geothermal, and hydro energy).^2,3^ Besides waste heat, ubiquitous heat sources such as the sun or even human bodies are also present. Therefore, a technology for the conversion of low-grade heat into electricity is highly desirable and can largely contribute to reducing the ongoing energy crisis. In this regard, thermoelectric (TE) devices have been considered as a potential power generation technology due to their capacity to directly convert heat into electricity in an eco-friendly, clean, and quiet manner.^4,5^ Current applications of TE technology include power generation from car exhausts, powering instruments for space exploration, heat recovery from industrial furnaces, solar power generation, sensor powering, and many more.^4–6^ Furthermore, TE devices also enable precise temperature control and active cooling across various technological and industrial applications.

TE material performance relies on the dimensionless figure of merit *zT* = *S* ^2^σ*T*/κ, where *S* is the Seebeck coefficient, σ is the electrical conductivity, *T* is the absolute temperature, and κ is the thermal conductivity. Thus, a high *zT* requires a large power factor (PF = *S* ^2^σ), and a low κ. Most of the current advancements in the figure of merit have been achieved mainly due to a significant reduction of κ through nanostructuring, doping, or defect engineering. However, κ is nearly reaching its amorphous limit, and further *zT* improvements, still necessary, require strategies to improve the PF.^7,8^ Nevertheless, PF improvement strategies are less common and challenging due to the inverse *S*–σ relationship.^9–11^

A few years ago, our group reported large PF improvements in a novel solid–liquid hybrid system consisting of a porous and nanostructured TE solid (Sb-doped SnO_2_) that was contacted with a liquid electrolyte. That study showed that using a solution of lithium tetrafluoroborate (LiBF_4_) in 3-methoxypropionitrile (3-MPN) as electrolyte, a 3.4 times PF enhancement can be produced in the Sb/SnO_2_ film. Additionally, using the ionic liquid 1-butyl-3-methylimidazolium iodide (BMII) as electrolyte, 2.4 times PF improvements were achieved.^12^ Based on this strategy, we have also reported similar improvements in this system when a redox molecule or a polymer electrolyte is used as the liquid.^13,14^ However, although we have produced significant PF improvements in Sb/SnO_2_, the obtained PF values were low, since Sb/SnO_2_ exhibits modest TE properties (*S* ≈ 45 μV/K).

Here, we investigate whether the PF enhancements can also be produced for a high performing n-type TE oxide. Among the oxide-based semiconductors, ZnO is the best candidate, since it shows one of the best performances.^15,16^ Moreover, ZnO is relatively cheap and safe^16,17^ compared to Te-based materials, such as Bi_2_Te_3_ and PbTe.^18,19^

To prove PF improvements in this oxide, first, we have prepared a porous and nanostructured ZnO material combined with Ag nanoparticles to improve its electrical conductivity. Then, the Ag–ZnO composite was permeated with two ionic liquids: (i) 1-butyl-3-methylimidazolium iodide (BMII) and (ii) 1-butyl-3-methylimidazolium bis­(trifluoromethylsulfonyl)­imide (BMITFSI). The variations in the PF in both cases were evaluated, and the changes found were investigated by scanning electron microscopy (SEM), X-ray diffraction (XRD), energy-dispersive X-ray spectroscopy (EDX), and impedance spectroscopy.

## Experimental Section

### Preparation of Porous Ag–ZnO Pellets

To produce the Ag–ZnO composite nanopowder, 200 mL of a 10 M NaOH (Sigma-Aldrich) solution was prepared in a 500 mL container. Due to the highly exothermic nature of NaOH dissolution, a container larger than the solution volume was used, and the lid was kept open to prevent pressure buildup. Immediate and continuous stirring after water addition was essential to avoid aggregation and ensure complete dissolution. In a large beaker placed inside a fume hood, 11.01 g (0.06 mol) of zinc acetate [Zn­(OAc)_2_] (Sigma-Aldrich) and 8 g (0.042 mol) of citric acid (Sigma-Aldrich) were weighed and combined with 200 mL of deionized water and 40 mL of ethanol (Sigma-Aldrich). A large magnetic stir bar was used to ensure effective mixing, as smaller bars may not adequately stir the increasingly viscous solution. The mixture was stirred until the solids dissolved, with intermittent sonication applied to accelerate dissolution.

Subsequently, 100 mg of silver nitrate (AgNO_3_) (Sigma-Aldrich) was added and dissolved. A 10 M NaOH solution was then added gradually to the vigorously stirred mixture until the pH reached 13. During this process, a white precipitate formed. After each addition of NaOH, the mixture was allowed to redistribute the forming solid before further additions. As more NaOH was added, the solution initially thickened, then became more fluid at higher pH levels. At near pH 13, additions were slowed to better control the end point without requiring dropwise precision. Once the pH reached 13, the resulting dispersion was transferred into hydrothermal reactors (Canrd, Ke Mi, China), filling each reactor to approximately three-quarters of its total volume. The reactors were sealed and heated in an oven at 150 °C for 15 h to carry out the hydrothermal reaction. After cooling, the resulting solid product was purified through 2–3 cycles of precipitation and redispersion using ethanol and water as solvents. To minimize material loss during high-temperature treatments, the solid was partially dried before calcination, preventing rapid evaporation and splattering during furnace processing. The dried material was then heated in air at 500 °C for 2–3 h. A slow temperature ramp was applied to accommodate residual moisture and reduce thermal stress. Following this step, the calcined solid was ground using a mortar to increase surface area and improve the efficiency of subsequent reduction. Finally, the powder was annealed under a reducing atmosphere (Ar/H_2_ mixture) at 500 °C for 2 h to complete the synthesis.

In order to prepare the Ag–ZnO pellets, a hot-pressing process was carried out in a glovebox filled with argon. 0.18 g of the Ag–ZnO powder was loaded into a graphite die with a square hole of 5 mm × 10 mm and sintered into pellets using a custom-made hot press. The hot-press sintering temperature, pressure and time of all pellets were 500 °C, 60 MPa and 8 min, respectively.

### Measurement System

In order to measure the PF of the pellets before and after their exposure to the different ionic liquids, a cell was constructed. It was formed by soda-lime glasses of 25 mm × 15 mm and 1.8 mm thickness as substrates, which were cleaned and UV–ozone treated following the procedure described elsewhere.^13^ Then, the as-prepared hot-pressed Ag–ZnO pellets were stuck on the glass substrates using a two-component transparent Poxipol epoxy adhesive applied laterally (see Figure 1). The pellets were then contacted at their ends by Ag paint (RS, ref 186-3600). Moreover, a total of 4 points were painted with the same Ag paint at the sides of the pellet, where two points were used to contact voltage probes, and the other two, aligned with the previous ones, to place thermocouples (see Figure 1).

**Figure 1:**
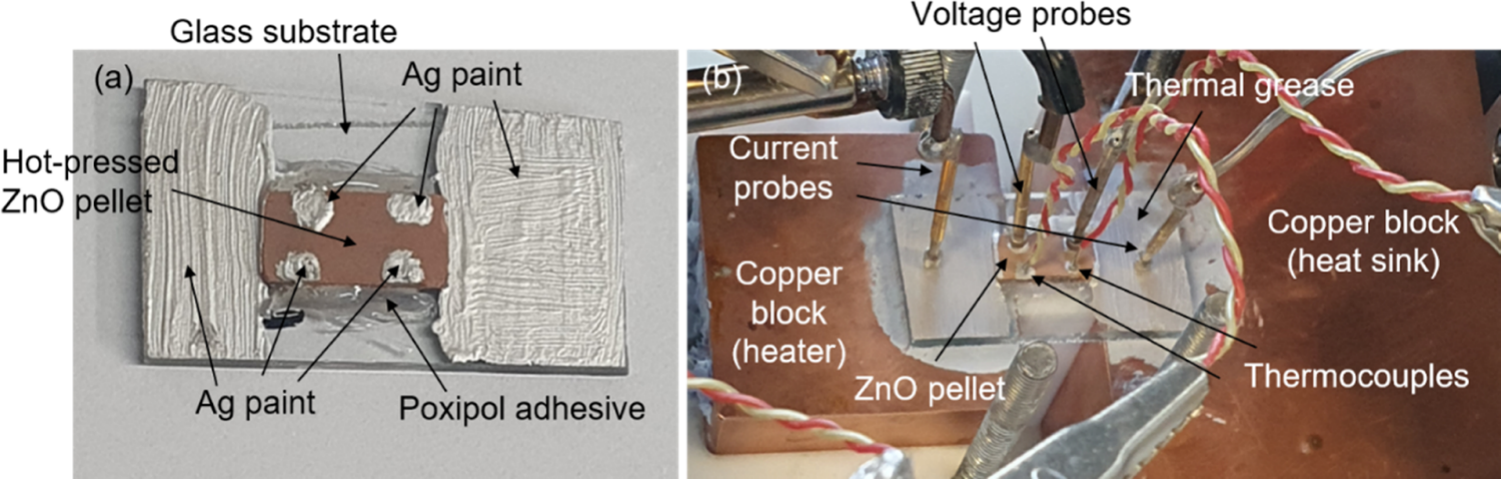
Images of (a) one of the Ag–ZnO cells prepared, and (b) a cell in the measurement setup contacted by different probes and thermocouples.

Once the cells were prepared, their initial PF was measured. After that, the cells were immersed in one of the two ionic liquids used: BMI (Solvionic, ref IM0406C10) or BMITFSI (Solvionic, ref IM0408A10) for 2 h, then rinsed with isopropanol (Labkem, PROL-P0P-5K0), and finally dried overnight. All these steps took place in a glovebox under N_2_ flow. After this treatment, the PF was measured again to identify the changes. One sample and a replica were used for each ionic liquid to check repeatability.

To measure the PF, the Seebeck coefficient and the electrical conductivity were determined using a homemade setup. As shown in Figure 1b, two copper blocks with different dimensions were used in this setup to produce a temperature difference Δ*T*. A small copper block (30 mm × 30 mm × 10 mm) with 3 cartridge heaters (Watlow, ref C1E13-L12) inserted acted as the heat source. On the other hand, a large copper block (50 mm × 50 mm × 30 mm) was used as the heat sink. The prepared cell was placed on top of the copper blocks in such a way that one side lied on the heater, and the other side on the heat sink. A polytetrafluoroethylene (PTFE) bridge was used in order to separate both copper blocks. Thermal grease (RS, ref 2173835) was applied at the glass/copper interfaces to enhance the thermal contact and improve heat transfer.

In order to carry out the Seebeck coefficient measurement, the 4 points painted with Ag paint at the sides of the pellet were used. Two points were used for voltage probes (RS, ref 2615092) to measure the open-circuit voltage *V*_oc_, and the other two to place two K-type thermocouples (RS, ref 8140134) to measure the temperature differences, as shown in Figure 1b. These Ag point contacts were needed since the pellets were quite brittle and many times, the direct contact was difficult since the gentle pressure produced by the probes and the thermocouples cracked or damaged the sample at that contact zone, making the contact very difficult. To determine the Seebeck coefficient, *V*_oc_ was monitored at the same time that the temperature difference was varied. *S* was calculated from the slope of the *V*_oc_ vs Δ*T*. The temperature was recorded using a dual datalogger thermometer (RS, ref 1316). A nanovoltmeter (Keithley 2182A) was employed to measure the *V*_oc_. The error of the Seebeck coefficient measurements, calculated from the linear fitting error, was below 6%.

To perform the electrical conductivity measurements, the electrical resistance *R* was measured first in a 4-probe mode, using the same probes that recorded the *V*_oc_ in the Seebeck coefficient measurement for the voltage, and two probes positioned on the lateral Ag paint contacts for the current *I* (see Figure 1b). The electrical resistance was determined from the slope of current–voltage *I*–*V* curve under a Δ*T* = 0 K and scanning the current with 1 ms delay time with a Keithley 2450 source meter. The electrical conductivity σ = *L*/(*RA*) was then calculated using the distance between the voltage probes *L*, and the width and thickness of the pellets that provided the area *A*. The error of the electrical resistance measurements, obtained from the linear fitting error, was below 3%.

### Characterization

The crystalline structure of the samples was analyzed by XRD patterns obtained from a Bruker AXS D8 Advance X-ray diffractometer with Ni-filtered (2 μm thickness) Cu Kα radiation (λ = 1.5406 Å) operated at 40 mA and 40 kV. SEM images of the Ag–ZnO pellets and their chemical composition were obtained using a JEOL 7001F instrument (Oxford instrument) along with EDX.

Impedance spectroscopy measurements were carried out at Δ*T* = 5 K before and after the different treatments using the same configuration of the probes that were used to determine the electrical resistance. Measurements were performed in galvanostatic mode at 0 A direct current (DC) in the 10 mHz-90 kHz frequency range with 20 μA amplitude. A Metrohm Autolab PGSTAT204 instrument, equipped with a FRA32 M frequency response analyzer was used to conduct impedance measurements.

All the PF and impedance spectroscopy measurements were carried out in a glovebox under N_2_ flow, maintaining the relative humidity below 20% in all cases. This is to avoid the ionic liquids capturing a significant amount of water from the atmosphere.

## Results and Discussion

The XRD patterns of the Ag–ZnO nanopowder and the corresponding hot-pressed pellets (Figure 2) show distinct diffraction peaks associated with the hexagonal wurtzite ZnO phase (JCPDS 01-079-2205) and the face-centered cubic (fcc) metallic Ag phase (JCPDS 00-004-0783). The notably higher intensity of the Ag peaks in the pellet suggests enhanced Ag segregation and particle growth during the sintering process. The presence of well-defined peaks for both phases, without significant peak broadening or shifts, confirms the coexistence of the two crystalline components with minimal structural distortion, confirming the successful formation of a composite structure with integrated Ag nanoparticles within the ZnO matrix.

**Figure 2:**
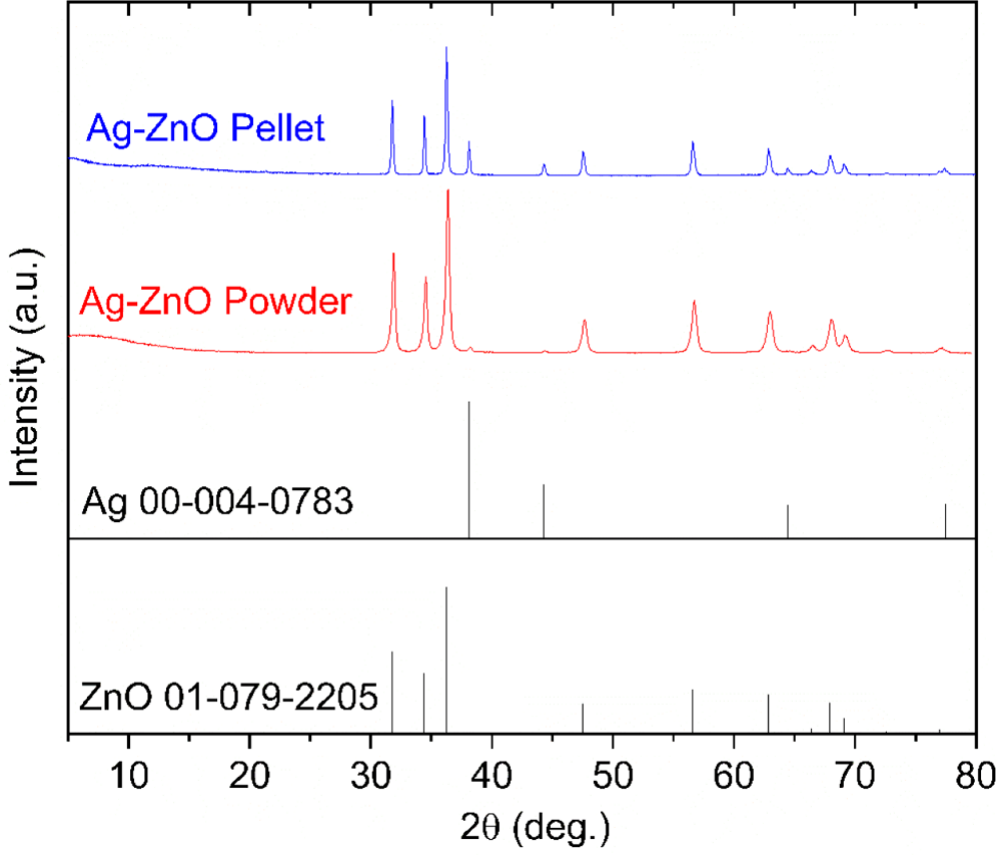
XRD patterns of the Ag–ZnO powder and an Ag–ZnO pellet. The reference fcc Ag and wurtzite ZnO peaks are also displayed as a reference.

Top-view and cross-sectional SEM images of the Ag–ZnO pellets (Figure 3) reveal a network of interconnected, rounded nanoparticles with diameters ranging from approximately 100 to 600 nm. The relatively mild conditions employed during the hot-pressing process effectively preserved the nanoparticulate morphology and prevented extensive sintering, resulting in a porous microstructure. This retained porosity is essential to enable subsequent infiltration with ionic liquid.

**Figure 3:**
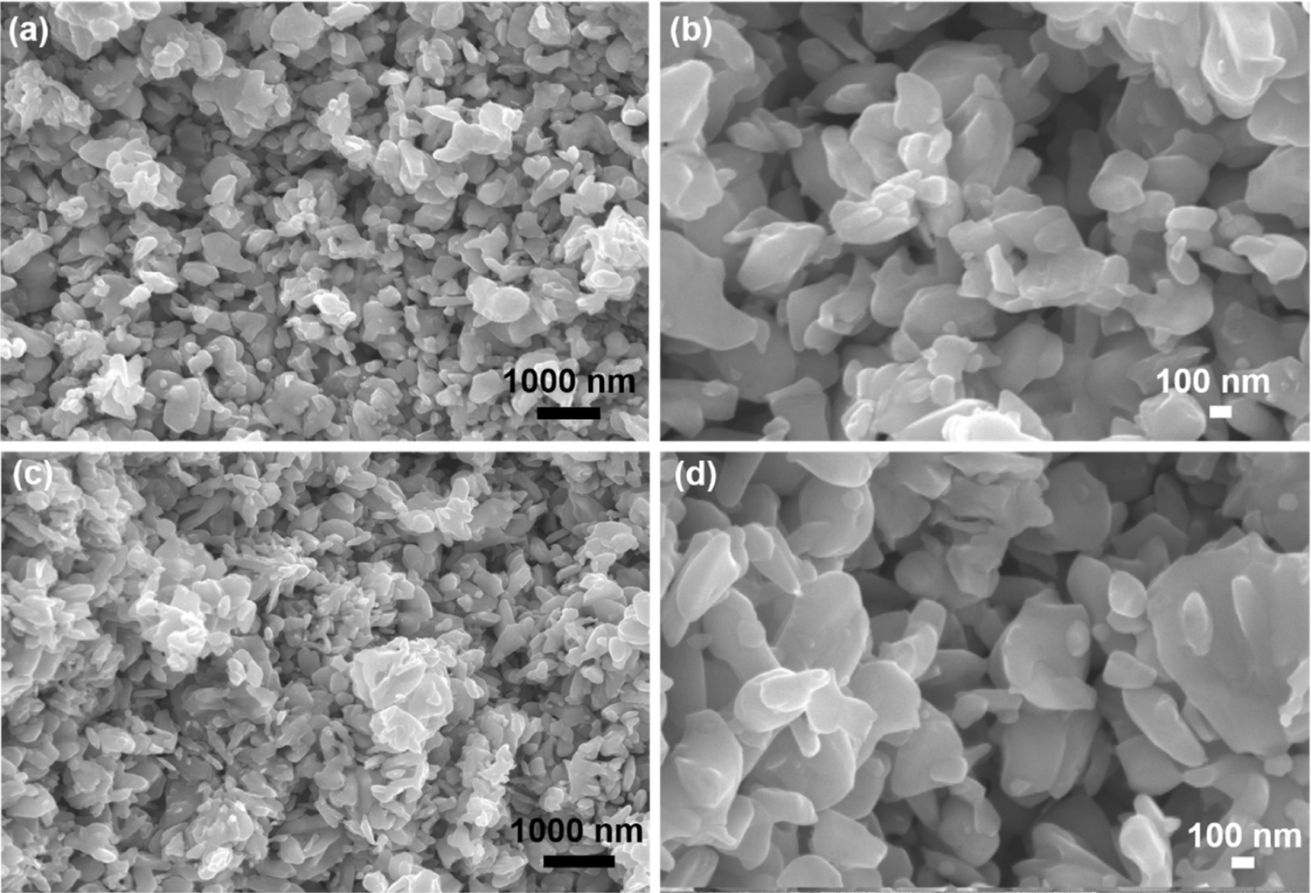
(a,b) Top-view and (c,d) cross-sectional SEM images of a Ag–ZnO pellet. (b,d) Show higher magnification views of (a,c), respectively.

The Ag–ZnO pellets were integrated into the cells and infiltrated with ionic liquids, as described in the Experimental Section. To assess the influence of the ionic liquids on the TE properties of Ag–ZnO, variations in the Seebeck coefficient and electrical conductivity were measured before and after each treatment. The summarized results are presented in Table 1, while the corresponding measurement data are provided in the Supporting Information (Figures S1, S2 and Table S1). We would like to remark that other electrolytes were also analyzed, but since they did not provide suitable results they have not been included in this study. However, they are reported here.^20^

**Table 1:** Seebeck Coefficient and Electrical Conductivity (and Their Variations) of Ag–ZnO Samples before (Ag–ZnO) and after Their Treatment with the Different Ionic Liquids [Ag–ZnO/BMIX (X = I/TFSI)]^a^

sample	Seebeck coefficient (μV/K)			electrical conductivity (S/m)			PF_after_/PF_before_
	Ag–ZnO	Ag–ZnO/BMIX	variation (%)	Ag–ZnO	Ag–ZnO/BMIX	variation (%)	
S1-BMII	–207.95	–184.59	–11.23	31.80	57.34	80.31	1.42
S2-BMII	–186.81	–180.51	–3.37	57.00	91.25	60.08	1.50
S1-BMITFSI	–179.98	–173.41	–3.65	9.93	11.21	12.89	1.05
S2-BMITFSI	–189.41	–183.81	–2.95	35.07	33.03	–5.81	0.89

a Also, the *PF* ratio (after and before the presence of the ionic liquids) is shown.

An initial average *S* value of 191.03 μV/K at room temperature was obtained for the as-prepared Ag–ZnO pellets, which is comparable, or even higher, than values reported in previous studies for this material. On the other hand, the average σ value at room temperature was 33.45 S/m, which is considerably lower than values reported in the literature.^17,21,22^ This reduced conductivity is attributed to the high porosity of the material and the large number of grain boundaries, which hinder charge carrier transport. However, these same structural features also contribute to a significant reduction in thermal conductivity, partially offsetting the impact on overall TE performance.

As shown in Table 1, the samples treated with BMII (S1-BMII and S2-BMII) show a slight reduction (7.3% average) in the absolute value of the Seebeck coefficient, accompanied by a significant enhancement in the electrical conductivity (70.2% average increase), leading to an average PF improvement of 1.46 times. It should be noted that in this system, a remarkably high increase in the electrical conductivity with a very small variation in the Seebeck coefficient is produced, which is highly desirable and not very frequent. Although this PF enhancement is not as high as in previously reported studies that employed Sb-doped SnO_2_ (around 3 times),^13,14^ the average PF value of Ag–ZnO/BMII is 2.47 μW/(K^2^m), which is ∼9.5 times higher than the PF value [0.26 μW/(K^2^m)] reported in one of the previous studies based on Sb/SnO_2_. This is mainly due to the higher *S* value of the Ag–ZnO (∼−185 μV/K), compared to the *S* value of Sb/SnO_2_ (∼−45 μV/K).^14^ On the other hand, although the Ag–ZnO/BMII PF value of 2.47 μW/(K^2^ m) is low compared with a highly compacted (nonporous) ZnO material, the thermal conductivity of the Ag–ZnO/BMII system is expected to be considerably reduced as mentioned above, which could lead to an overall suitable performance. We have not included thermal conductivity measurements in our study due to the complexity of our system (highly britlle ZnO material and hygroscopic ionic liquid), focusing our analysis on the PF.

Unlike the results obtained with BMII, when the Ag–ZnO pellets were combined with BMITFSI (S1-BMITFSI and S2-BMITFSI), almost no variation either in the absolute value of the Seebeck coefficient (3.3% average reduction) or in the electrical conductivity (3.54% average reduction) was found, resulting in no PF improvements. Since the only difference between the two ionic liquids is their anion (I^–^ and TFSI^–^), it is clear that the presence of I^–^ plays a key role in achieving the PF improvement.

To identify the origin of the PF variations found, the different systems tested were analyzed by XRD, SEM (EDX) and impedance spectroscopy. In particular, XRD patterns were acquired for the S1-BMII and S2-BMITFSI samples to determine whether the ionic liquid treatments induced changes in the crystalline phases. As shown in Figure 4, all patterns exhibit the characteristic peaks of the hexagonal wurtzite ZnO structure^23^ and the fcc Ag phase, consistent with the results presented earlier (Figure 2). Additional diffraction peaks are observed in the ionic liquid-treated samples (Ag–ZnO/BMII and Ag–ZnO/BMITFSI); however, these peaks are attributed to the presence of Ag paint applied on the sample surface (see Figure 1a), which was used for electrical contact in the treated samples but not in the untreated Ag–ZnO pellet. Therefore, the XRD analysis confirms that the crystalline structure of the Ag–ZnO pellets remains unchanged after ionic liquid infiltration.

**Figure 4:**
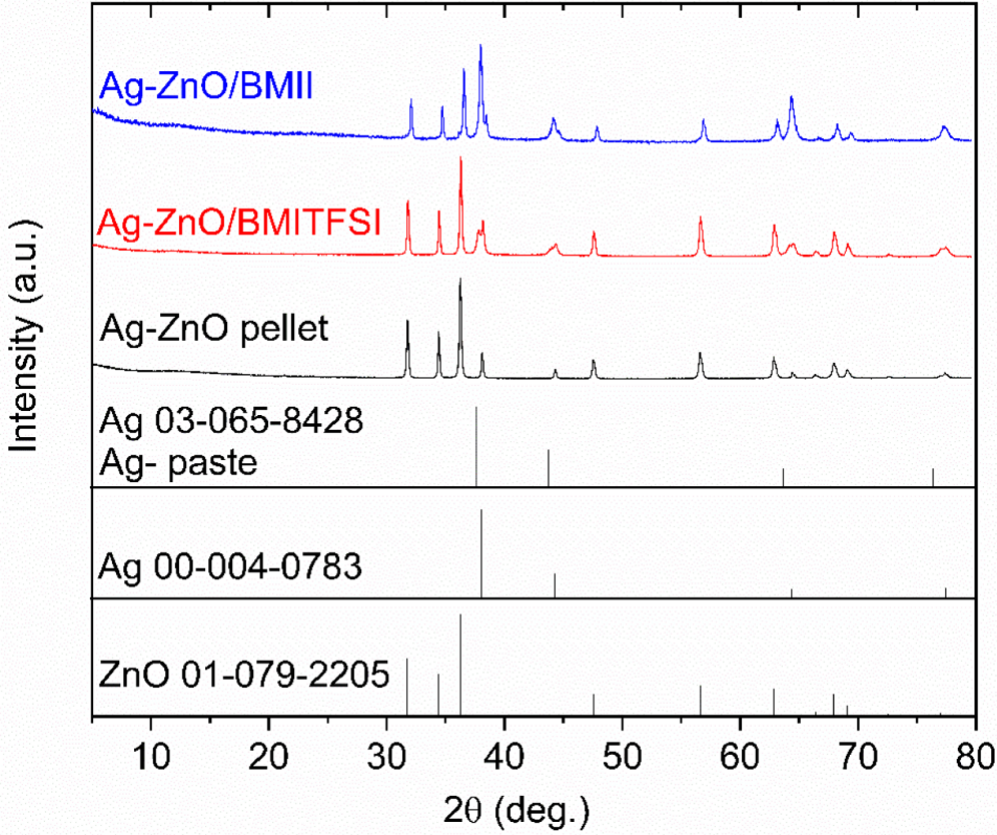
XRD patterns of Ag–ZnO, Ag–ZnO/BMII (S1-BMII) and Ag–ZnO/BMITFSI (S2-BMITFSI) along with the references of Ag, Ag paste, and ZnO.

To analyze possible changes in the surface morphology of the Ag–ZnO systems, before and after the treatments with the ionic liquids, SEM and EDX characterization was conducted. Figure 5 displays the top (a–c) and cross-sectional (d–f) SEM images of an untreated Ag–ZnO pellet, Ag–ZnO/BMII (S1-BMII), and Ag–ZnO/BMITFSI (S1-BMITFSI). Both top-view and cross-sectional images revealed no significant variation in the morphology of the samples. However, in the samples treated with the ionic liquids, it is evident that the liquids still impregnate the Ag–ZnO matrix, indicating that they were not completely removed during the washing and drying process. This was confirmed by the EDX elemental composition analysis shown in Table 2. It can be observed that apart from the expected elements (Ag, Zn, and O), C and I are present in the BMII-treated sample (S1-BMII), and C, N, F and S exist in the BMITFSI-treated sample, in agreement with the SEM images. Hence, the treated systems really behave as a solid–electrolyte system, where the ionic liquids (electrolytes) coat the Ag–ZnO solid surface.

**Figure 5:**
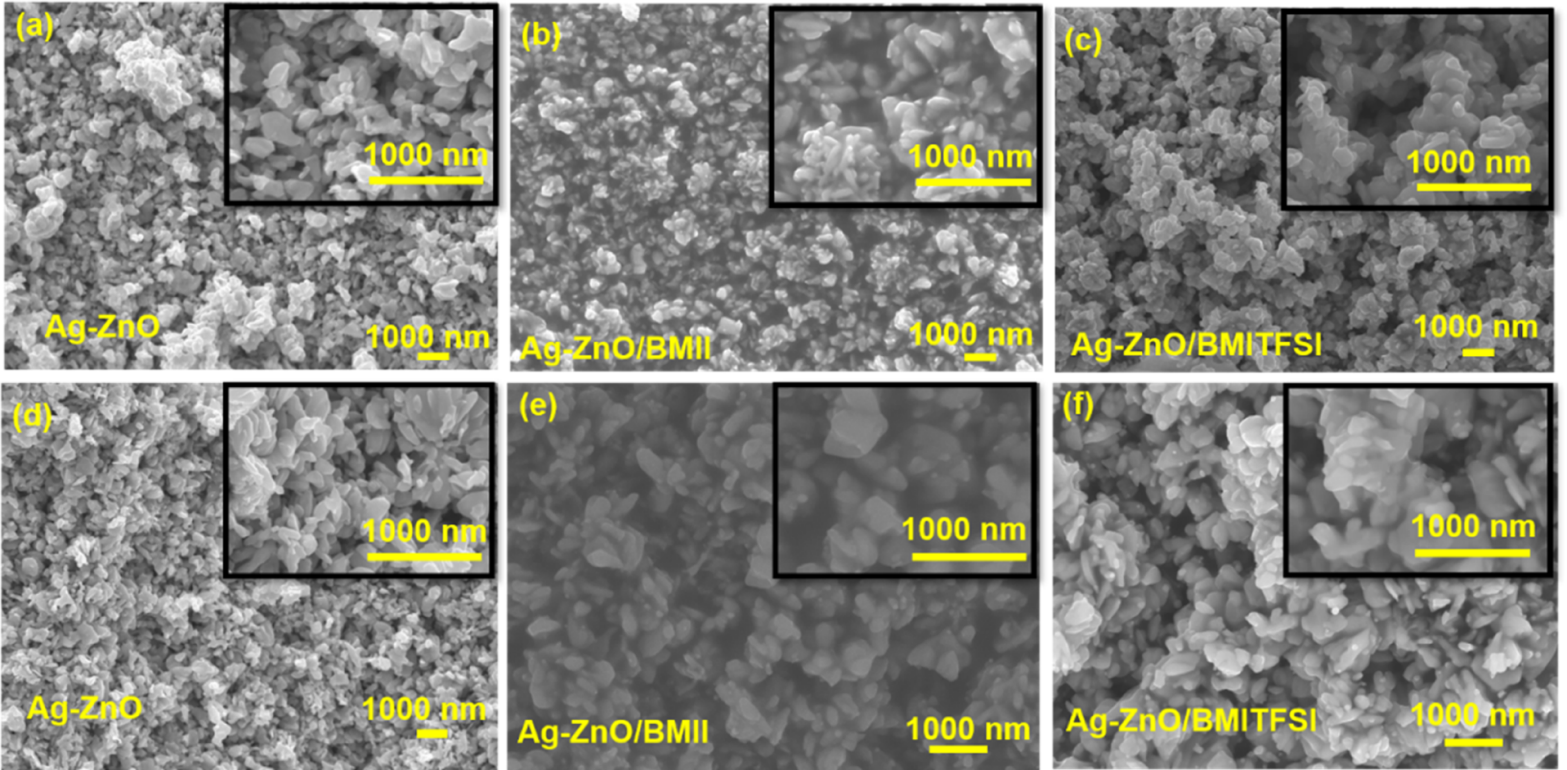
(a–c) Top-view and (d–f) cross-sectional SEM images of Ag–ZnO, Ag–ZnO/BMII (S1-BMII), and Ag–ZnO/BMITFSI (S1-BMITFSI). The insets show magnifications of each image.

**Table 2:** EDX Analysis Results of Ag–ZnO, Ag–ZnO/BMII (S1-BMII), and Ag–ZnO/BMITFSI (S1-BMITFSI) Samples^a^

	elements (at. %)							
sample	Zn	O	Ag	I	C	N	F	S
Ag–ZnO	45.11	54.05	0.84	-	-	-	-	-
S1-BMII	20.15	31.63	0.34	2.21	45.67	-	-	-
S1-BMITFSI	30.20	37.17	0.78	-	20.87	4.80	4.91	1.28

a EDX analysis was performed from the top-view SEM images of Figure 5.

Impedance spectroscopy analysis was performed to see if additional processes occur in the TE response of the treated samples. The results obtained for S1-BMII and S2-BMITFSI systems are shown in Figure 6. It can be observed that the impedance spectra only show points lying around certain real impedance (*Z*′) values in all cases, which is the hallmark of an ohmic behavior, and no additional features appear after the treatments, such as new semicircles, capacitive vertical rises, or diffusion (Warburg-like) trends. It should be noted that the values of the ohmic resistance from Figure 6 are in good agreement with the results shown in Table S1. The lack of additional features leads to the conclusion that the electronic transport along the oxide film is the mechanism that govern the response in all cases, which is determined by the mobility and concentration of the carriers (electrical conductivity). The mobility of the carriers is not expected to vary significantly, since the morphology of the oxide films do not show notable changes after being impregnated with the ionic liquids (Figure 5). Hence, the variation of the carrier concentration of the film is the only possible reason behind the electrical conductivity increase in the samples impregnated with BMII. Note that although ionic liquids are good ionic conductors, they do not allow the electrical conduction.

**Figure 6:**
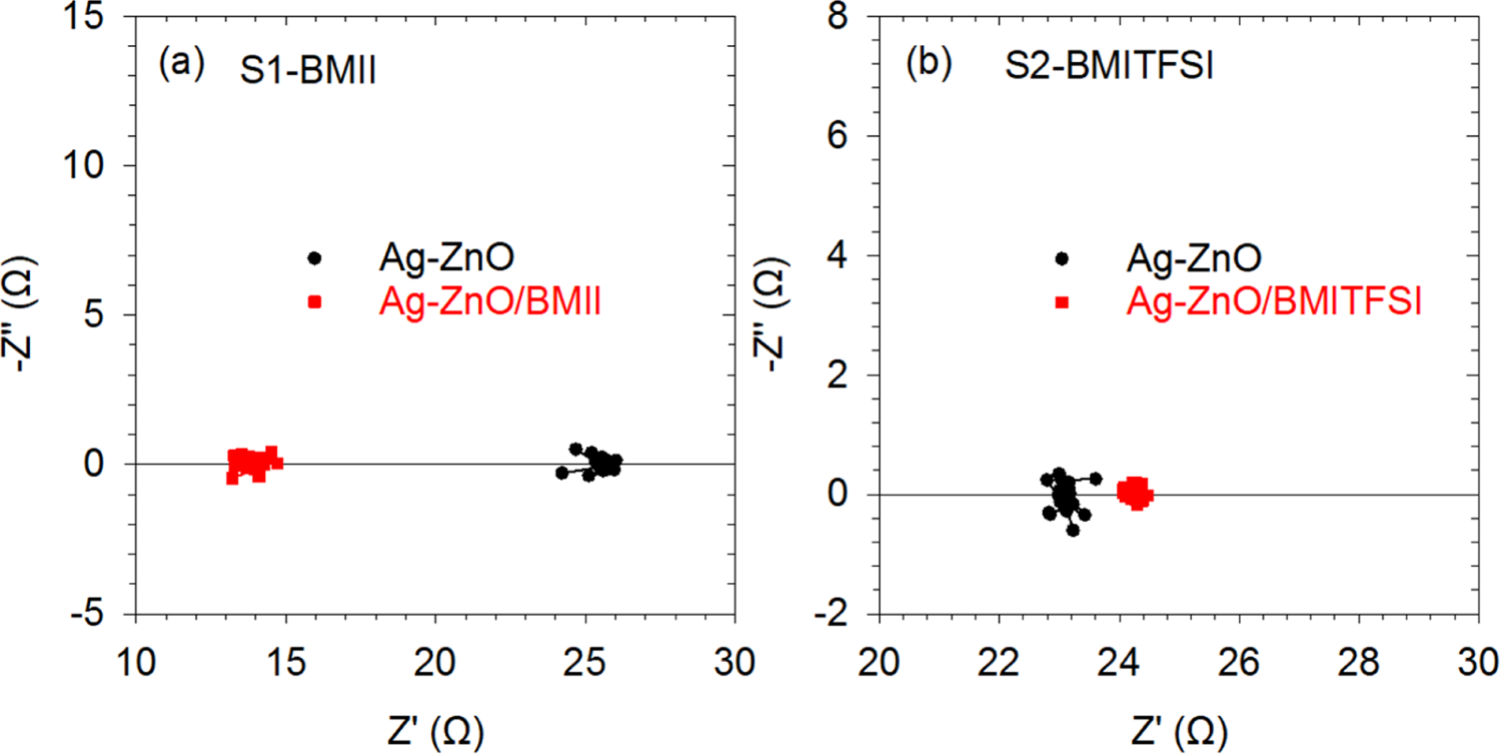
Impedance spectroscopy spectra carried out at Δ*T* = 5 K for Ag–ZnO pellets before (circles) and after (squares) being exposed to (a) BMII and (b) BMITFSI. The spectra were measured for samples S1-BMII (a) and S2-BMITFSI (b).

The carrier concentration increase for the samples treated with BMII can be achieved due to an injection of electrons from the I^–^ ions present in the ionic liquid covering the Ag–ZnO. A similar mechanism was attributed in previous studies to electrical conductivity improvements in Sb-doped SnO_2_ in contact with electrolytes^13,14^ or functionalized with redox species.^24^ This increase in the electrical conductivity remarkably takes place without seriously affecting the Seebeck coefficient.

Unlike TFSI, I^–^ is a redox ion, which means that can exchange electrons with the oxide. More specifically, since it is a reductant, it can inject electrons (oxidants accept electrons). Examples of nanostructured and porous ZnO films that exchange electrons with iodide ions can be found i.e. in dye-sensitized solar cells, which use the iodide/triodide redox couple.^25^ The variation of the carrier concentration *n* by injection of electrons from redox species in nanostructured and porous oxides, such as ZnO, is possible since they typically show an exponential density of states below the conduction band edge energy level *E*_CB_, which is due to existence of a large number of surface and trap states, as well as interparticle boundaries.^26,27^ If the carrier concentration is low (e.g., 10^18^ to 10^19^ cm^–3^),^26,27^ the Fermi level *E*_F_ typically lies within this exponential density of states and can move closer to the conduction band when exchanging electrons (equilibrating) with redox species of an electrolyte, increasing thus the carrier concentration in the material and hence the electrical conductivity, as schematically shown in Figure 7.

**Figure 7:**
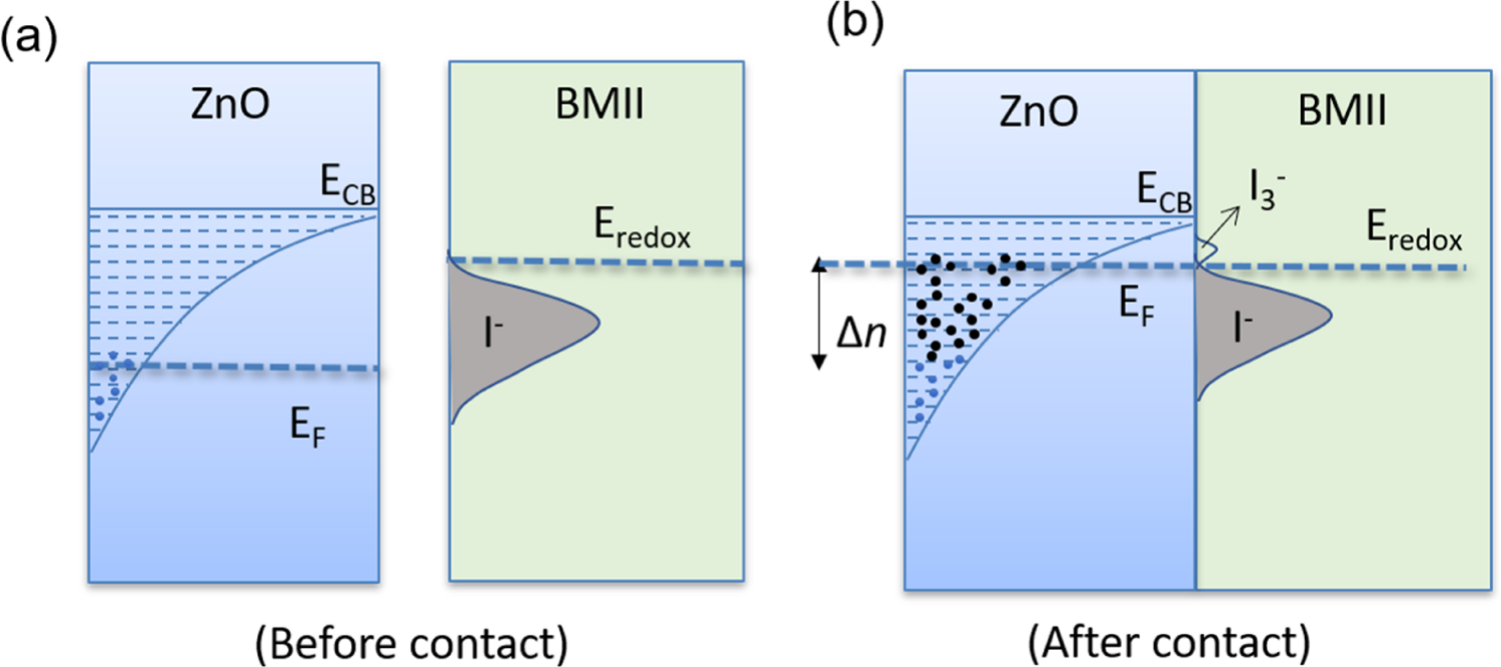
Diagrams of the energetic situations (a) before and (b) after the contact of the Ag–ZnO films with the BMII ionic liquid.

The carrier concentration of the Ag–ZnO film (before being in contact with any ionic liquid) was measured by Hall effect (see Figure S3), obtaining a carrier concentration value of ∼5 × 10^18^ cm^–3^, which lies in the carrier concentration range mentioned above, where the Fermi level can be shifted by equilibration with redox species.

## Conclusions

In this study, we have analyzed power factor improvements in porous ZnO when it is contacted with different ionic liquids. First, we prepared ZnO nanoparticles combined with Ag by a hydrothermal method that led to a Ag–ZnO composite with good thermoelectric properties. The particles were partially compacted by hot press to obtain porous pellets that were brought into contact with two ionic liquids [1-butyl-3-methylimidazolium iodide (BMII) and 1-butyl-3-methylimidazolium bis­(trifluoromethylsulfonyl)­imide (BMITFSI)]. The treatment with the ionic liquid BMII led to a significant rise in the electrical conductivity (70.2% average), with only an average 7.3% decrease in the absolute value of the Seebeck coefficient, leading to a 1.46 times average PF enhancement. In contrast, no PF improvement was achieved when BMITFSI was tested. An XRD analysis showed no structural modifications of the treated samples. On the other hand, SEM images and XRD revealed that the ionic liquids remained coating the Ag–ZnO matrix after the treatments, with no alteration of the surface morphology. Finally, impedance spectroscopy experiments found no additional features in the impedance response, concluding that an ohmic response occurs in all cases. Under these observations, the electrical conductivity increase found for BMII was attributed to the injection of charge from the I^–^ ions of BMII in the oxide, leading to an increase in the carrier concentration. This study introduces a new route to increase the electrical conductivity of ZnO with nearly no variation in its Seebeck coefficient, which is not very common and highly beneficial.

## Supplementary Material



## Notes

Seebeck coefficient and electrical resistance measurements; electrical conductivity determination; Hall effect measurements (PDF)

## References

[1] Bianchi G., Panayiotou G. P., Aresti L., Kalogirou S. A., Florides G. A., Tsamos K., Tassou S. A., Christodoulides P. (2019). Estimating
the Waste Heat Recovery in the European Union Industry. Energy, Ecol. Environ..

[2] Forman C., Muritala I. K., Pardemann R., Meyer B. (2016). Estimating the Global
Waste Heat Potential. Renew. Sustain. Energy
Rev..

[3] Firth A., Zhang B., Yang A. (2019). Quantification
of Global Waste Heat
and Its Environmental Effects. Appl. Energy.

[4] Beretta D., Neophytou N., Hodges J. M., Kanatzidis M. G., Narducci D., Martin-Gonzalez M., Beekman M., Balke B., Cerretti G., Tremel W., Zevalkink A., Hofmann A. I., Müller C., Dörling B., Campoy-Quiles M., Caironi M. (2019). Thermoelectrics: From
History, a
Window to the Future. Mater. Sci. Eng., R.

[5] Champier D. (2017). Thermoelectric
Generators: A Review of Applications. Energy
Convers. Manag..

[6] Ando
Junior O. H., Maran A. L. O., Henao N. C. (2018). A Review of the
Development and Applications of Thermoelectric Microgenerators for
Energy Harvesting. Renew. Sustain. Energy Rev..

[7] Wu Z., Zhang S., Liu Z., Mu E., Hu Z. (2022). Thermoelectric
Converter: Strategies from Materials to Device Application. Nano Energy.

[8] Zhou W. X., Cheng Y., Chen K. Q., Xie G., Wang T., Zhang G. (2020). Thermal Conductivity of Amorphous
Materials. Adv. Funct. Mater..

[9] Dun C., Hewitt C. A., Huang H., Xu J., Zhou C., Huang W., Cui Y., Zhou W., Jiang Q., Carroll D. L. (2015). Flexible N-Type Thermoelectric Films Based on Cu-Doped
Bi2Se3 Nanoplate and Polyvinylidene Fluoride Composite with Decoupled
Seebeck Coefficient and Electrical Conductivity. Nano Energy.

[10] Mehdizadeh
Dehkordi A., Zebarjadi M., He J., Tritt T. M. (2015). Thermoelectric
Power Factor: Enhancement Mechanisms and Strategies for Higher Performance
Thermoelectric Materials. Mater. Sci. Eng.,
R.

[11] Lin Z., Ping X., Zhao D., Wang L., Li M., Cai Z., Zhang Y., Li X., Zhang X. (2023). Construct Schottky
Interface Containing Energy-Filtering Effect: An Efficient Strategy
to Decouple Thermopower and Conductivity. J.
Appl. Phys..

[12] Márquez-García L., Beltrán-Pitarch B., Powell D., Min G., García-Cañadas J. (2018). Large Power Factor Improvement in
a Novel Solid–Liquid Thermoelectric Hybrid Device. ACS Appl. Energy Mater..

[13] Castro-Ruiz S., Márquez-García L., Solis-De la Fuente M., Beltrán-Pitarch B., Mota-Babiloni A., Vidan F., Íñigo-Rabinal P., Guisado-Barrios G., García-Cañadas J. (2023). Power Factor
Improvement in a Solid-Liquid Thermoelectric System Formed by Sb:SnO2
in Contact with a Chromium Complex Solution. Sustain. Energy Fuels.

[14] Solis-De
la Fuente M., Castro-Ruiz S., Márquez-García L., Rullière P., Fantini S., Del Olmo R., Casado N., García-Cañadas J. (2025). Poly­(Diallyldimethylammonium)-Based
Solid Electrolytes to Significantly Enhance the Power Factor of a
Thermoelectric Oxide Film (Sb-Doped SnO2). Sustain.
Energy Fuels.

[15] Nam W. H., Lim Y. S., Choi S. M., Seo W. S., Lee J. Y. (2012). High-Temperature
Charge Transport and Thermoelectric Properties of a Degenerately Al-Doped
ZnO Nanocomposite. J. Mater. Chem..

[16] Zhu B. B., Li D., Zhang T. S., Luo Y. B., Donelson R., Zhang T., Zheng Y., Du C. F., Wei L., Hng H. H. (2018). The Improvement
of Thermoelectric Property of Bulk ZnO via ZnS Addition: Influence
of Intrinsic Defects. Ceram. Int..

[17] Wu Z. H., Xie H. Q., Zhai Y. B. (2015). Preparation
and Thermoelectric Properties
of Co-Doped ZnO Synthesized by Sol-Gel. J. Nanosci.
Nanotechnol..

[18] Brintha S. R., Ajitha M. (2015). Synthesis and Characterization of ZnO Nanoparticles
via Aqueous Solution, Sol-Gel and Hydrothermal Methods. IOSR J. Appl. Chem..

[19] Rani K., Gupta V., Ranjeet, Pandey A. (2023). Improved Thermoelectric Performance
of Se-Doped n-Type Nanostructured Bi2Te3. J.
Mater. Sci.:Mater. Electron..

[20] UncorrelaTEd project . Deliverable 3.2: Optimum Electrolytes to Maximise the Power Factor in the Porous Oxide Materials (v2), 2024, URL: Https://Cordis.Europa.Eu/Project/Id/863222/Results (accessed 15 01, 2026).

[21] Cheng H., Xu X. J., Hng H. H., Ma J. (2009). Characterization of
Al-Doped ZnO Thermoelectric Materials Prepared by RF Plasma Powder
Processing and Hot Press Sintering. Ceram. Int..

[22] Biswas S., Singh S., Singh S., Chattopadhyay S., De Silva K. K. H., Yoshimura M., Mitra J., Kamble V. B. (2021). Selective
Enhancement in Phonon Scattering Leads to a High Thermoelectric Figure-of-Merit
in Graphene Oxide-Encapsulated ZnO Nanocomposites. ACS Appl. Mater. Interfaces.

[23] Colder H., Guilmeau E., Harnois C., Marinel S., Retoux R., Savary E. (2011). Preparation of Ni-Doped ZnO Ceramics
for Thermoelectric
Applications. J. Eur. Ceram. Soc..

[24] Rahman M. M., Márquez-García L., Solis-De la Fuente M., García-Cañadas J. (2025). Remarkable
Power Factor Improvement
in a Porous, Nanostructured Thermoelectric Oxide Functionalized with
Viologen Molecules. Sustain. Energy Fuels.

[25] Anta J. A., Guillén E., Tena-Zaera R. (2012). ZnO-Based Dye-Sensitized Solar Cells. J. Phys. Chem. C.

[26] Bisquert J., Fabregat-Santiago F., Mora-Seró I., Garcia-Belmonte G., Barea E. M., Palomares E. (2008). A Review of Recent Results on Electrochemical
Determination of the Density of Electronic States of Nanostructured
Metal-Oxide Semiconductors and Organic Hole Conductors. Inorg. Chim. Acta.

[27] Fabregat-Santiago F., Mora-Seró I., Garcia-Belmonte G., Bisquert J. (2003). Cyclic Voltammetry
Studies of Nanoporous Semiconductors. Capacitive and Reactive Properties
of Nanocrystalline TiO2 Electrodes in Aqueous Electrolyte. J. Phys. Chem. B.

