# Supplementary material for: Improvement of the Thermoelectric Power Factor of ZnO Using Ionic Liquids
Source: ACS Appl Electron Mater. 2026 Jan 29;8(3):1061–8. doi: 10.1021/acsaelm.5c01462 (PMC12895412; doi:10.1021/acsaelm.5c01462)
Supplement: Supplementary file 1 [file el5c01462_si_001.pdf]

Supporting information

Improvement of the thermoelectric power factor of ZnO using ionic liquids

Md Mahmudur Rahman[1+], Lourdes Márquez-García[1+], Guillem Montaña-Mora[2], Ke Xiao[2], Mauricio Solis-de la Fuente[1], Sergio Castro-Ruiz[1], Sébastien Fantini[3], Andreu Cabot[2,4], Jorge García-Cañadas[1*]

[1] Universitat Jaume I, Av. Vicent Sos Baynat s/n, 12003 Castelló de la Plana, Spain.
[2] Institut de Recerca en Energia de Catalunya (IREC), Jardins de les Dones de Negre 1, 08930 Sant Adrià de Besòs, Catalonia, Spain.
[3] Solvionic, 11 Chem. des Silos, 31100 Toulouse, France.
[4] ICREA, Pg. Lluis Companys, Barcelona 08010, Catalonia, Spain.
[+] These authors contributed equally
**Corresponding author e-mail: garciaj@uji.es*

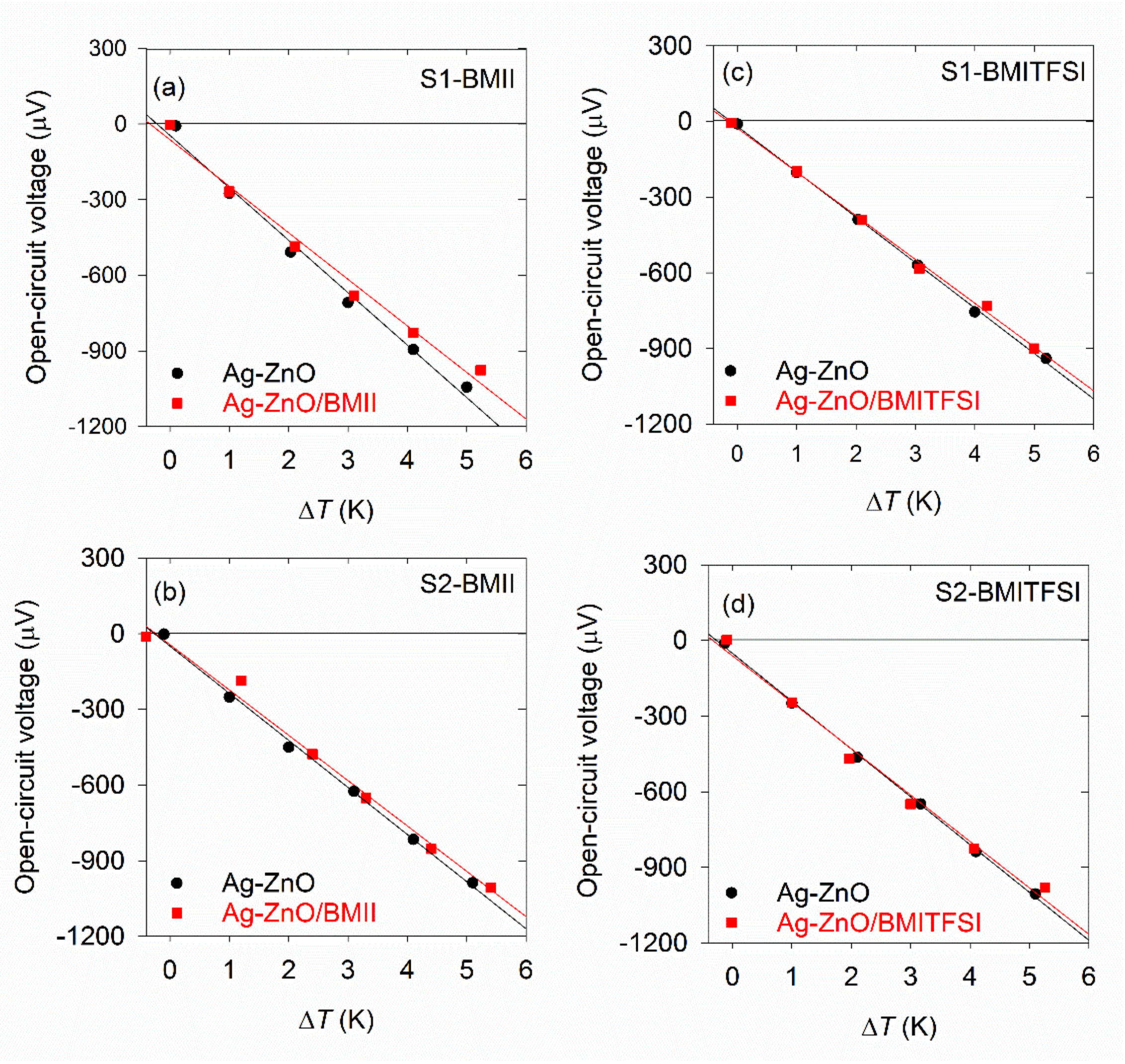

Fig. S1. Open-circuit voltage vs temperature difference curves for the determination of the Seebeck coefficient before (Ag-ZnO) and after [Ag-ZnO/BMIX (X=I/TFSI)] the treatments with the different ionic liquids. Lines correspond to the linear fits.

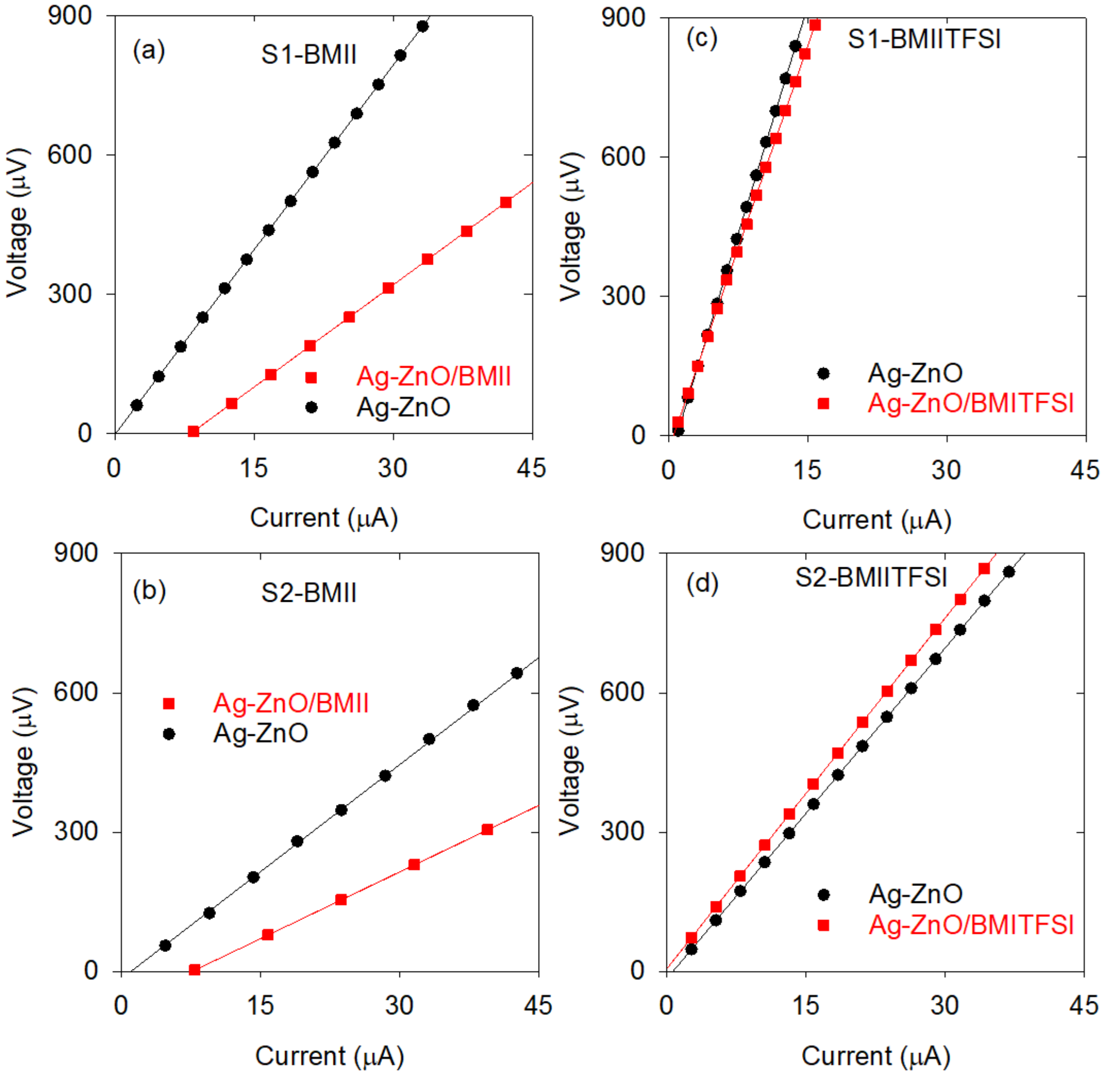

Fig. S2. Voltage vs current curves for the determination of the electrical resistance before (Ag-ZnO) and after [Ag-ZnO/BMIX (X=I/TFSI)] the treatments with the different ionic liquids. Lines correspond to the linear fits.

Table S1. Details for the calculation of the electrical conductivity of Ag-ZnO pellets before (Ag-ZnO) and after [Ag-ZnO/BMIX (X=I/TFSI)] their treatment with the ionic liquids.

Sample	Thickness (mm)	Length (mm)	Width (mm)	Resistance (Ω)		Electrical conductivity (S/m)	
				Ag-ZnO	Ag-ZnO/BMIX	Ag-ZnO	Ag-ZnO/BMIX
S1-BMII	1.04	4.26	4.86	26.50	14.70	31.80	57.34
S2-BMII	1.10	5.30	5.50	15.37	9.60	57.00	91.25
S1-BMITFSI	1.15	4.00	5.35	65.47	58.00	9.93	11.21
S2-BMITFSI	1.02	4.72	5.56	23.73	25.20	35.07	33.03

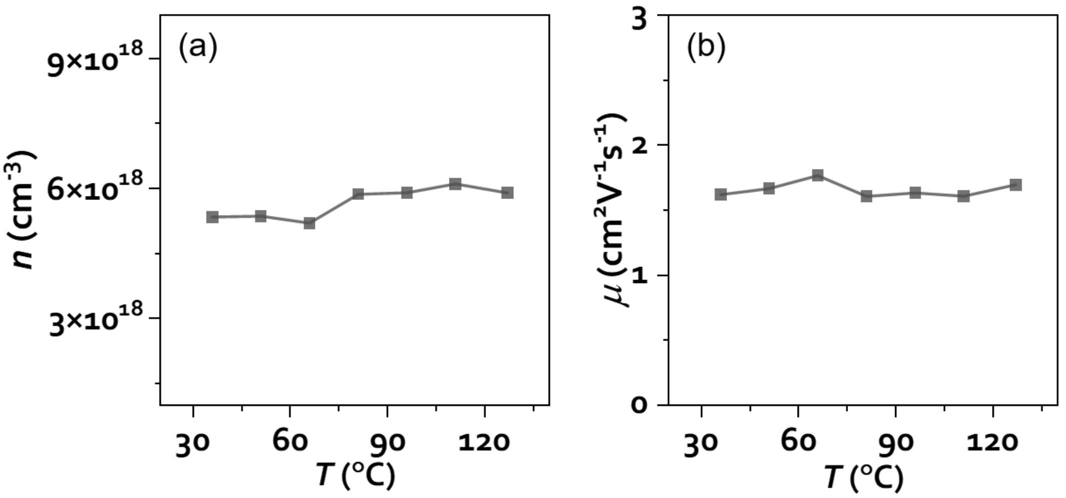

Fig. S3. (a) Carrier concentration and (b) mobility as a function of temperature for a Ag-ZnO film. The Hall coefficient was measured using a Linseis HCS 10 system at a current of 20 mA and under a magnetic field ranging from −0.5 T to 0.5 T, from which the Hall carrier concentration and Hall mobility were subsequently calculated.
